# Hochuekkito (TJ-41), a Kampo Formula, Ameliorates Cachexia Induced by Colon 26 Adenocarcinoma in Mice

**DOI:** 10.1155/2012/976926

**Published:** 2012-12-24

**Authors:** Suzu Yae, Fumiyuki Takahashi, Toshifumi Yae, Takuji Yamaguchi, Rika Tsukada, Kengo Koike, Kunihiko Minakata, Akiko Murakami, Fariz Nurwidya, Motoyasu Kato, Mayumi Tamada, Momoko Yoshikawa, Hiroyuki Kobayashi, Kuniaki Seyama, Kazuhisa Takahashi

**Affiliations:** ^1^Department of Respiratory Medicine, School of Medicine, Juntendo University, 2-1-1 Hongo, Bunkyo-ku, Tokyo 113-8421, Japan; ^2^Research Institute for Diseases of Old Age, School of Medicine, Juntendo University, 2-1-1 Hongo, Bunkyo-ku, Tokyo 113-8421, Japan; ^3^Center for Advanced Kampo Medicine and Clinical Research, School of Medicine, Juntendo University, 2-1-1 Hongo, Bunkyo-ku, Tokyo 113-8421, Japan; ^4^Division of Gene Regulation, Institute for Advanced Medical Research, School of Medicine, Keio University, 35 Shinanomachi, Shinjyuku-ku, Tokyo 160-8582, Japan

## Abstract

Cachexia, a major cause of cancer-related death, is characterized by depletion of muscle and fat tissues, anorexia, asthenia, and hypoglycemia. Recent studies indicate that secretions of proinflammatory cytokines such as interleukin-6 (IL-6) play a crucial role in cachexia development, and that these cytokines are secreted from not only cancer cells but also host cells such as macrophages. In this study, we investigated the therapeutic effects of hochuekkito, a Kampo formula, on cachexia induced by colon 26 adenocarcinoma in mice. Hochuekkito treatment did not inhibit tumor growth, but significantly attenuated the reduction in carcass weight, food and water intake, weight of the gastrocnemius muscle and fat tissue around the testes, and decrease of serum triglyceride level compared with controls. Furthermore, hochuekkito treatment significantly reduced serum IL-6 level and IL-6 expression level in macrophages in tissues surrounding the tumor. In vitro studies showed that hochuekkito suppressed the production of IL-6 by THP-1 or RAW264.7 macrophage cells, although it did not affect IL-6 production by colon 26 carcinoma cells. These results suggest that hochuekkito inhibits the production of proinflammatory cytokines, particularly IL-6, by host cells such as macrophages. Therefore, hochuekkito may be a promising anticachectic agent for the treatment of patients with cancer.

## 1. Introduction

 Cancer cachexia, which is characterized by the loss of muscle and fatty tissue as well as anorexia, asthenia, and anemia, makes therapeutic interventions difficult in cancer patients [1, 2]. Cachexia is associated not only with deterioration of the quality of life (QOL) but also with shorter survival times [1, 2]. Therefore, it is important to manage the cachectic state in cancer patients. However, current therapies for cancer cachexia are of limited benefit due to poor efficacy and multiple side effects [3]. The establishment of a new therapeutic modality to prevent the cachectic condition with few side effects is required for cancer patients with cachexia. 

 Although the precise mechanisms of cancer cachexia are not fully elucidated, it is now clear that proinflammatory cytokines, such as interleukin-6 (IL-6) and tumor necrosis factor-*α* (TNF-*α*), which are derived from both tumor cells or host cells, are involved in induction and development of cancer cachexia [4, 5]. These cytokines have been demonstrated to cause metabolic alterations, resulting in the loss of muscle and adipose tissues in the host [6]. Among them, IL-6 is considered to be a key mediator in the pathogenesis of cancer cachexia. In cancer patients, high levels of circulating IL-6 were observed with almost every type of tumor and predicted a poor outcome [7]. In experimental animal models, treatment with monoclonal antibody to IL-6 or IL-6 receptor significantly suppressed the development of cachexia in tumor-bearing mice [8, 9]. We recently reported that tocilizumab, humanized monoclonal antibody against the IL-6 receptor, had the dramatic effect on cachexia in a patient with IL-6 overexpressing lung cancer [10]. These lines of evidence indicate that proinflammatory cytokines such as IL-6 play pivotal roles in the pathogenesis of cancer cachexia and are potential targets for the therapy of cancer patients with a cachectic condition. 

 Hochuekkito is a Kampo formula and is composed of 10 species of medicinal plants. It has been widely used for the treatment of complaints of general fatigue caused by common colds with few side effects. Hochuekkito improved the QOL and immunological status of elderly patients [11, 12]. In addition, treatment with hochuekkito ameliorated systemic inflammation and body weight loss and improved nutritional status and QOL of patients with chronic obstructive pulmonary disease (COPD) [13]. 

These previous reports prompted us to investigate the efficacy of hochuekkito in cachexia. Here, we examined the effects of hochuekkito on the severity of key parameters of cachexia in well-established murine model of experimental cachexia induced by colon 26 (C26) adenocarcinoma. We also investigated the effects of hochuekkito on the production of proinflammatory cytokines including IL-6 from C26 adenocarcinoma cells and two macrophage cell lines in vitro, and discussed the significance of hochuekkito in treatment of cancer cachexia.

## 2. Materials and Methods

### 2.1. Animals

Virus-free male BALB/c mice were purchased from Japan SLC (Hamamatsu, Japan) at 5 to 6 weeks of age. The animals were group-housed (5 per cage) at a temperature of 24 ± 2°C, relative humidity of 55% ± 10%, and a 12 h light/12 h dark cycle. After habituation for 1 week, mice were housed 2 per cage throughout the experiment. Chlorinated water and irradiated food were provided ad libitum. All procedures performed on the animals were approved by the Institutional Animal Care and Use Committee (IACUC) of Juntendo University, approval number 220010.

### 2.2. Drugs

**Figure 1:**
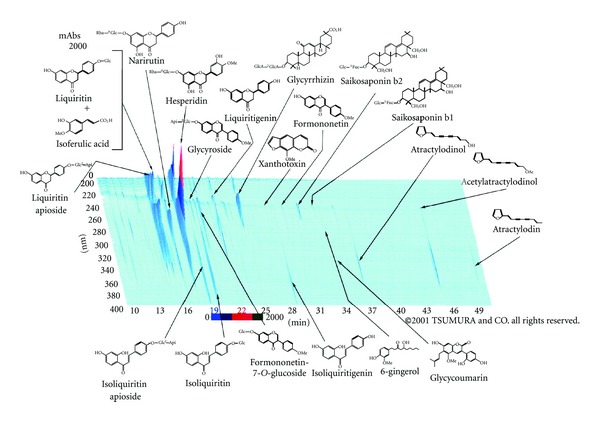
Three-dimensional HPLC profile of hochuekkito. Each peak in the HPLC profile of hochuekkito was identified by comparison with the retention times and UV spectra of chemically defined standard compounds. HPLC conditions were column: Tosoh TSK GEL ODS-80Ts (4.6 × 250 mm), carrier A: 0.05 M ammonium acetate (pH 3.6), carrier B: acetonitrile, gradient: linear 10–100% carrier B in 60 min, flow rate: 1.0 mL min^−1^, and injection volume: 30 *μ*L. Detector: Shimadzu SPD-M10A VP.

Hochuekkito (TJ-41, Lot. no. 2100041010) was kindly supplied from Tsumura Co. (Tokyo, Japan). Hochuekkito is manufactured as a spray-dried powder of hot water extract obtained from 10 medicinal plants in the following ratio: Astragalus Root (4.0, roots of *Astragalus membranaceus* Bunge), Atractylodes Lancea Rhizome (4.0, rhizomes of *Atractylodes lancea* DC.), Ginseng Radix (4.0, roots of *Panax ginseng* C.A. Meyer), Angelica Radix (3.0, roots of *Angelica acutiloba* Kitagawa), Bupleurum Radix (2.0, roots of *Bupleurum falcatum* L.), Zizyphi Fructus (2.0, fruits of *Zizyphus jujuba* Miller var. inermis Rehder), Aurantii Bobilis Pericarpium (2.0, pericarps of ripe fruits of *Citrus unshu* Markovich), Glycyrrhizae radix (1.5, roots of *Glycyrrhiza uralensis *Fisch et DC.), Cimicifugae Rhizome (1.0, rhizomes of *Cimicifuga simplex* Worms kjord), and Zingiberis Rhizoma (0.5, rhizomes of *Zingiber officinale* Roscoe). The quality of some component indicators of this drug is controlled by measuring the contents by high performance liquid chromatography (HPLC). Chemical profile of Hochuekkito obtained by the 3D HPLC analysis is shown in Figure 1.

### 2.3. Cell Lines

Colon 26 (C26) adenocarcinoma (clone 20) with a potent ability to induce lethal cachexia in mice was kindly provided by the laboratory of Chugai Pharmaceutical Co. (Kamakura, Japan) [14]. Human macrophage cell line THP-1 was obtained from the American Type Culture Collection (ATCC) (Manassas, VA, USA). Murine macrophage cell line, RAW264.7 cells, was purchased from Riken Cell Bank (Tsukuba, Japan). Cells were maintained in RPMI 1640 (Wako, Osaka, Japan) with 10% fetal bovine serum (FBS) and penicillin and streptomycin (100 U/mL and 100 *μ*g/mL, resp.). 

### 2.4. Mouse Models of Cachexia

C26 (clone 20) cells (1 × 10^6^) were subcutaneously (s.c.) inoculated into the right flank of each mice. Tumor growth was measured twice a week using a digital caliper and recorded as the longest surface length (a) and width (b) in cm. Tumor volume (*V*, cm^3^) was calculated according to the following formula: *V* = *ab* ^2^/2. Mice were randomly assigned to 4 groups. Group 4 received the standard diet mixed with hochuekkito at a dose of 1% (i.e., 1.2 g/kg body weight) [15, 16] from days 0 to 14 after tumor injection. This group was labeled Tumor (+), hochuekkito (+). To dissect effects peculiar to hochuekkito on cachexia, the diet same as that administered to group 4 was administered to another group of mice (group 2), and this group was labeled Tumor (−), hochuekkito (+), and another group of tumor-bearing mice was administered the normal diet (group 3; this group was labeled Tumor (+), hochuekkito (−)). The healthy control group included age-matched mice without any treatment (group 1; this group was labeled Tumor (−), hochuekkito (−)). Group 1 and 2 consisted of 5 mice, and other groups consisted of 4 mice. All experiments were performed twice. During time-course experiments, the mice were carefully monitored and body weight and food/water intake were measured. Fifteen days after tumor inoculation, the mice were anesthetized with somnopentyl and euthanized. At the time of sacrifice, the tumor, gastrocnemius muscle, and fat tissue around the testes were dissected and weighed [17, 18]. The carcass weight was calculated as the difference between the weight of the whole body and the tumor as described previously [14]. The tumor weight was estimated from the tumor volume (*V* = *ab* ^2^/2) by multiplying this by a correction factor, which was determined by comparing actual tumor weights with tumor volumes [19]. Blood samples were collected, and the serum was separated within 1 h of sacrifice. Serum levels of IL-6 and tumor necrosis factor *α* (TNF-*α*) were measured by enzyme-linked immunosorbent assay (ELISA) using IL-6 and TNF-*α* mouse ELISA Kit (R&D Systems, Minneapolis, MN, USA). Additionally, to determine the nutritional status of the animals, we measured the serum levels of glucose, triglycerides, total cholesterol, hemoglobin (Hb), and hematocrit (Hct) and white blood cell (WBC) counts, red blood cell (RBC) counts, and platelet (Plt) count in blood using laboratory tests. 

### 2.5. Immunohistochemical Analysis

Tissue was fixed in 4% paraformaldehyde, embedded in paraffin, and cut into 4-*μ*m-thick sections. Sections were depleted of paraffin and then rehydrated in a graded series of ethanol solutions. For immunohistochemical analysis, serial sections in the same sample were used. Sections were washed with Tris-buffered saline (TBS), subjected to antigen retrieval by heating for 10 min at 100°C in 0.01 M sodium citrate (pH 6.0), and exposed to 3% H_2_O_2_ before incubation with primary antibodies, Mac-1/CD11b antibody (BD Pharmingen, NJ, USA) or IL-6 antibody (Abcam, Cambridge, UK). Immune complexes were detected with the use of a Vectastain Elite kit (Vector Laboratories, Burlingame, CA) and 3,3-diaminobenzidine, and sections were counterstained with hematoxylin. 

Areas containing Mac-1/CD11b-positive infiltrating macrophages were first identified by scanning tissue sections, and the macrophage count was determined in 4 such areas under ×200-magnification. The area positive for Mac-1/CD11b or IL-6 immunoreactivity was quantified with the use of BZ Analyzer software (Keyence, Osaka, Japan), using a constant color threshold in 4 fields per slide. IL-6 production by macrophages was estimated by the ratio of the IL-6 positive area to the Mac-1 positive area. 

### 2.6. Determination of the Inhibitory Effect of Hochuekkito on the Production of Proinflammatory Cytokines by Macrophages

THP-1 cells (5 × 10^5^ cells/well in 6 well plate) were cultured for 24, 48, or 72 h in the absence or presence of hochuekkito at a concentration of 0, 10, 50, 100, or 500 *μ*g/mL. The culture supernatants were collected by centrifugation, and the concentration of cytokines secreted into the supernatant was quantitated by using Multiplex Suspension Array (Genetic Lab Corp. Ltd., Sapporo, Japan) according to the manufacturer's instructions. RAW264.7 cells (5 × 10^5^ cells/6 cm dish) were stimulated with lipopolysaccharide (LPS) from *Escherichia coli *serotype 055:B5 (Sigma) (1 *μ*g/mL) [17] because the level of cytokines secreted from RAW264.7 cells was below the lower limit of detection without a stimuli for 24, 48, or 72 h in the absence or presence of hochuekkito at a concentration of 0, 10, 50, 100, and 500 *μ*g/mL [20]. The culture supernatants were collected, and the concentration of IL-6 and TNF-*α* secreted into the supernatant was quantitated by using ELISA according to the manufacturer's instructions.

### 2.7. In Vitro Cell Proliferation Assay

C26 carcinoma cells, RAW264.7 cells, or THP-1 cells were plated onto 96-well plates at 1 × 10^3^ cells/well in quadruplicate. Cells were grown in the absence or presence of hochuekkito at the concentrations of 0, 10, 50, 100, and 500 *μ*g/mL. At designated time points, the number of cells was quantified using Cell Counting Kit-8 (Wako, Japan) as described previously [21].

### 2.8. Statistics

Data are presented as means ± S.D. Variables for different groups were compared using unpaired *t*-test or Tukey's Honest Significant Differences (HSD) after analysis of variance (ANOVA); *P* < 0.05 was considered statistically significant. 

## 3. Results

### 3.1. Hochuekkito Improved Cachexia in Colon 26 Adenocarcinoma-Bearing Mice

The day when C26 clone 20 cells were inoculated into the mice was designated as day 0. All of the mice inoculated with clone 20 died between 16 and 20 days after tumor inoculation. We therefore evaluated cancer cachexia in the experimental model 15 days after tumor inoculation. At the end of experiments on day 14, weights of the carcass, gastrocnemius muscle, and fat tissue around the testes were significantly lower in untreated tumor-bearing mice (tumor (+) hochuekkito (−); group 3) than in healthy control mice (tumor (−) hochuekkito (−); group 1) (Table 1). In addition, the concentration of Hb, Hct value, Plt count, and the levels of triglyceride and glucose were significantly lower in the untreated tumor-bearing mice (group 3) than in the normal mice (group 1) (Table 1). 

**Figure 2:**
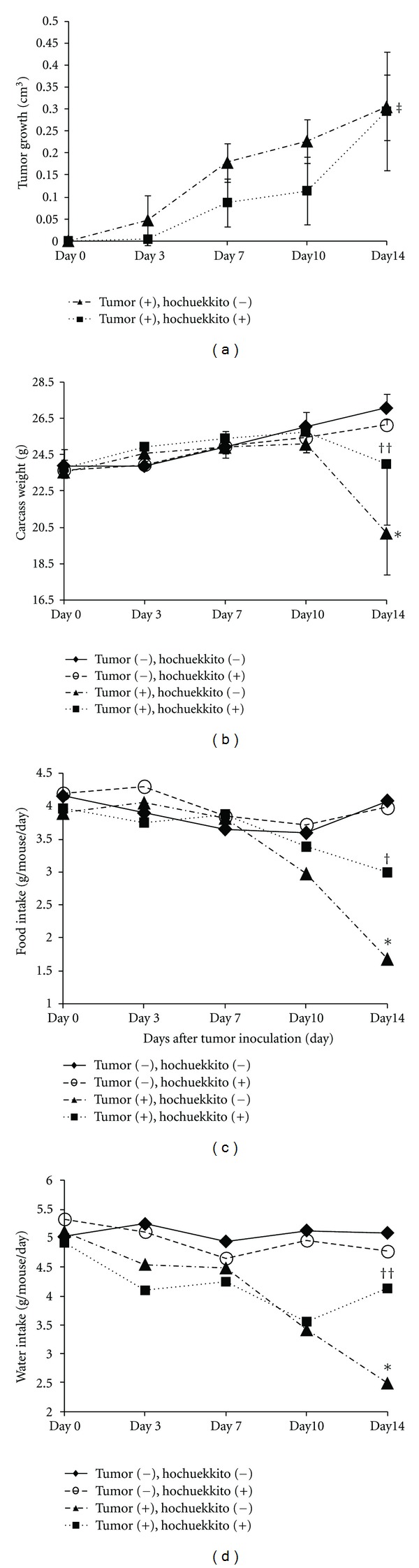
Effect of hochuekkito on tumor growth (a), carcass weight (b), food intake (c), and water intake (d) in colon 26/clone 20 adenocarcinoma-bearing mice. Tumor cells (1 × 10^6^) were implanted s.c. into mice on day 0, and hochuekkito administered orally to mice for consecutive 15 days (*⚪* tumor (−), hochuekkito (+); *n* = 5, ■ tumor (+), hochuekkito (+); *n* = 4) and no drug was given to another group (▲ tumor (+), hochuekkito (−); *n* = 4). The group tumor (−), hochuekkito (−) was healthy control mice (◆; *n* = 5). Tumor size, body weight, food intake, and water intake were measured on days 0, 3, 7, 10, and 14. The measured quantity of food or water was divided by the number of mice and days to determine each intake per animal per day. Animal experiments were performed twice, and a representative result is depicted. Data are presented as means ± SD. Statistical significance was evaluated with Tukey's Honest Significant Differences (HSD) after the one-way ANOVA. ^‡^No significant difference in tumor growth between hochuekkito (−) or hochuekkito (+) on day14. *Significantly different from the group of tumor (−), hochuekkito (−) in the same day; *P* < 0.01. ^†^Significantly different from the group of tumor (+), hochuekkito (−) in the same day; *P* < 0.01. ^††^Significantly different from the group of tumor (+), hochuekkito (−) in the same day; *P* < 0.05.

Hochuekkito was administered orally from the day after tumor inoculation and was continued for 15 consecutive days (tumor (+) hochuekkito (+); group 4). As shown in Figure 2(a), treatment with hochuekkito did not affect the growth rate of C26 adenocarcinoma in mice on day 14 (tumor (+) hochuekkito (−); group 3 versus tumor (+) hochuekkito (+); group 4). However, the decrease in the carcass weight in hochuekkito-treated tumor-bearing mice (group 4) was significantly smaller than that in the untreated tumor-bearing mice (group 3) (Table 1 and Figure 2(b)). Progressive reduction in food and water intake was observed in tumor-bearing mice beginning on day 7, and significant differences were observed in these values between hochuekkito-treated and untreated tumor-bearing mice on day 14 (Figures 2(c) and 2(d)). No significant differences were observed in the carcass weight and food and water intakes between untreated and treated healthy controls (Figures 2(b), 2(c), and 2(d)). These results indicate that administration of hochuekkito significantly decreased the reduction in carcass weight and food and water intakes without affecting tumor growth in a murine model of cachexia induced by C26 adenocarcinoma. 

No significant differences were observed in Hb concentration, Hct value, and Plt counts between hochuekkito-treated and untreated tumor-bearing mice on day 14. However, the serum triglyceride level was significantly higher in hochuekkito-treated mice than in the untreated mice (Table 1). In addition, we evaluated the weights of muscle and fat tissues in the murine model of cachexia. Weights of the gastrocnemius muscle and fat tissue around the testes in tumor-bearing mice treated with hochuekkito were significantly higher than those of untreated tumor-bearing mice (Table 1). No significant differences were observed in the serum triglyceride level and the weights of the gastrocnemius muscle and fat tissue around the testes between untreated and treated healthy controls (Table 1). 

### 3.2. Hochuekkito Inhibited Serum IL-6 Level in Colon 26 Adenocarcinoma-Bearing Mice

**Figure 3:**
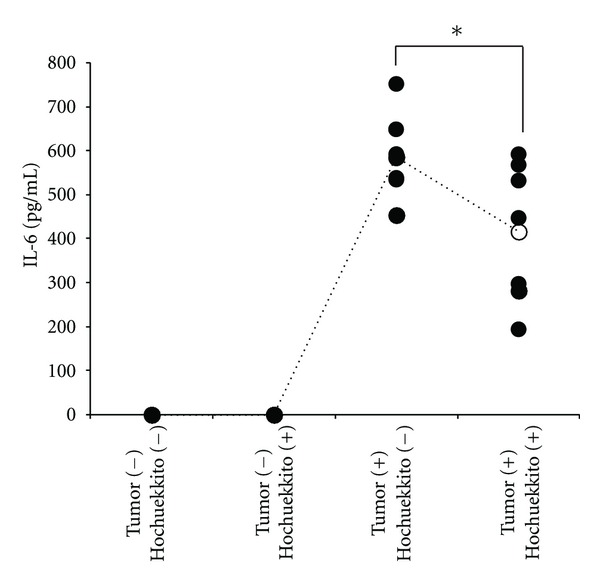
Effect of hochuekkito on serum IL-6 level in colon 26/clone 20 adenocarcinoma-bearing mice. Tumor cells (1 × 10^6^) were implanted s.c. into mice on day 0, and hochuekkito was administered orally to mice for consecutive 15 days. The serum was collected at the time of killing and measured by enzyme-linked immunosorbent assay (ELISA) using an IL-6 mouse ELISA kit. Number of serum samples is 7 for each group. Statistical significance was evaluated with Tukey's HSD after the one-way ANOVA. *Significantly different from the group of tumor (+), hochuekkito (−); *P* < 0.05.

The IL-6 and TNF-*α* levels in the serum were measured by ELISA. We confirmed that the serum level of IL-6 was below the lower limit of detection in healthy control mice, and IL-6 level was significantly upregulated in untreated tumor-bearing mice (Figure 3). Furthermore, serum IL-6 level was significantly decreased by the treatment with hochuekkito in tumor-bearing mice when compared to untreated mice (Figure 3). The serum level of TNF-*α* was below the lower limit of detection in all groups (data not shown). Taken together, these findings suggest that administration of hochuekkito significantly decreased the serum level of IL-6 in the tumor-bearing mice and attenuated the metabolic alterations in muscle and fat tissues associated with cancer cachexia. 

### 3.3. Effect of Hochuekkito on Macrophage Infiltration and IL-6 Production by Macrophages in Colon 26 Adenocarcinoma-Bearing Mice

**Figure 4:**
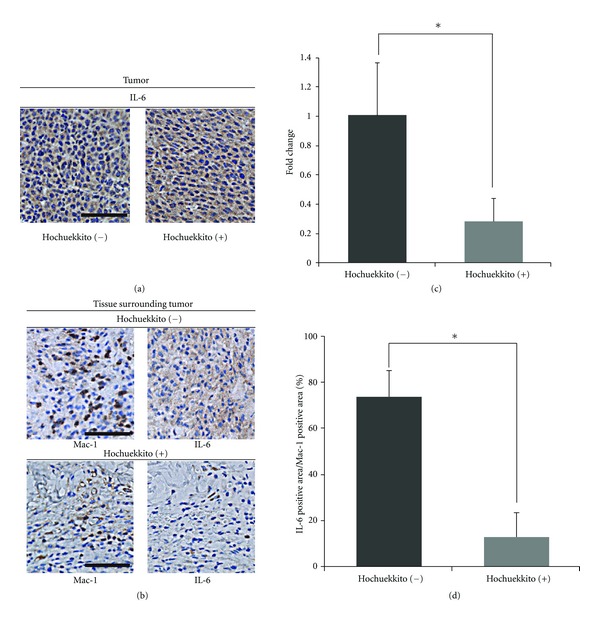
Effect of hochuekkito on macrophage infiltration and IL-6 production by macrophages or cancer cells from C26-bearing mice. (a) Immunostaining of IL-6 in tumors from C26-bearing mice that had been treated with or without hochuekkito, 15 days after inoculation of cancer cells. Scale bars: 100 *μ*m. (b) Immunostaining of Mac1/CD11b and IL-6 in tissues surrounding tumors from C26-bearing mice with or without treatment with hochuekkito, 15 days after inoculation of cancer cells. Serial sections from the same tissue sample were used for analysis. Scale bars: 100 *μ*m. (c) Quantitative immunohistochemical analysis of macrophage infiltration. The bar graph shows the fold-change in macrophage infiltration in tissues surrounding tumors in C26-bearing mice, treated with or without hochuekkito, calculated by setting the ratios of macrophage counts in untreated mice as 1. *Statistically significant difference compared with the group of hochuekkito (−); *P* < 0.01 (d) Quantitative immunohistochemical analysis for IL-6 production by macrophages in tissues surrounding tumors in C26-bearing mice treated with or without hochuekkito. The area positive for Mac-1 or IL-6 immunoreactivity was quantified in 4 fields per slide. IL-6 production by macrophages was estimated by determining the ratio of the IL-6-positive area to the Mac-1-positive area. Quantitative data are presented as means ± SD. Statistical significance was evaluated with unpaired Student's *t*-test. *Statistically significant difference compared with the group of hochuekkito (−); *P* < 0.01.

To further investigate the effect of hochuekkito in vivo, we examined expression of IL-6 in tumor tissues collected from C26-bearing mice treated with or without hochuekkito. At first, we performed immunohistochemistry to detect IL-6 and evaluated the inhibitory effect of hochuekkito on IL-6 production in tumor-bearing mice. IL-6 was highly expressed by cancer cells in tumor tissues from C26-bearing mice (Figure 4(a)). However, IL-6 expression level in cancer cells was not altered by the treatment with hochuekkito by immunohistochemical analysis (Figure 4(a)). Therefore, we also examined tissues surrounding tumors as microenvironment. For immunohistochemical analysis for IL-6 and macrophage-specific marker Mac-1, serial sections from the same tissue sample were used. Interestingly, IL-6 was also strongly expressed in macrophages in the tissues surrounding tumors without hochuekkito (Figure 4(b)). Quantitative immunohistochemical analysis demonstrated that infiltration of macrophages in the tissues surrounding tumors was significantly reduced by the administration with hochuekkito (Figures 4(b) and 4(c)), and no infiltrating macrophage was found within the C26 tumors (data not shown). IL-6 production by macrophages was estimated by the ratio of the IL-6 positive area to the Mac-1 positive area in the tissues surrounding tumors and was significantly inhibited by the treatment with hochuekkito (Figure 4(d)). Taken together, these findings suggest hochuekkito ameliorated experimental cancer cachexia in mice through the inhibition of macrophage-derived cytokine production, especially IL-6.

### 3.4. Hochuekkito Inhibits In Vitro IL-6 Production by Macrophages

**Figure 5:**
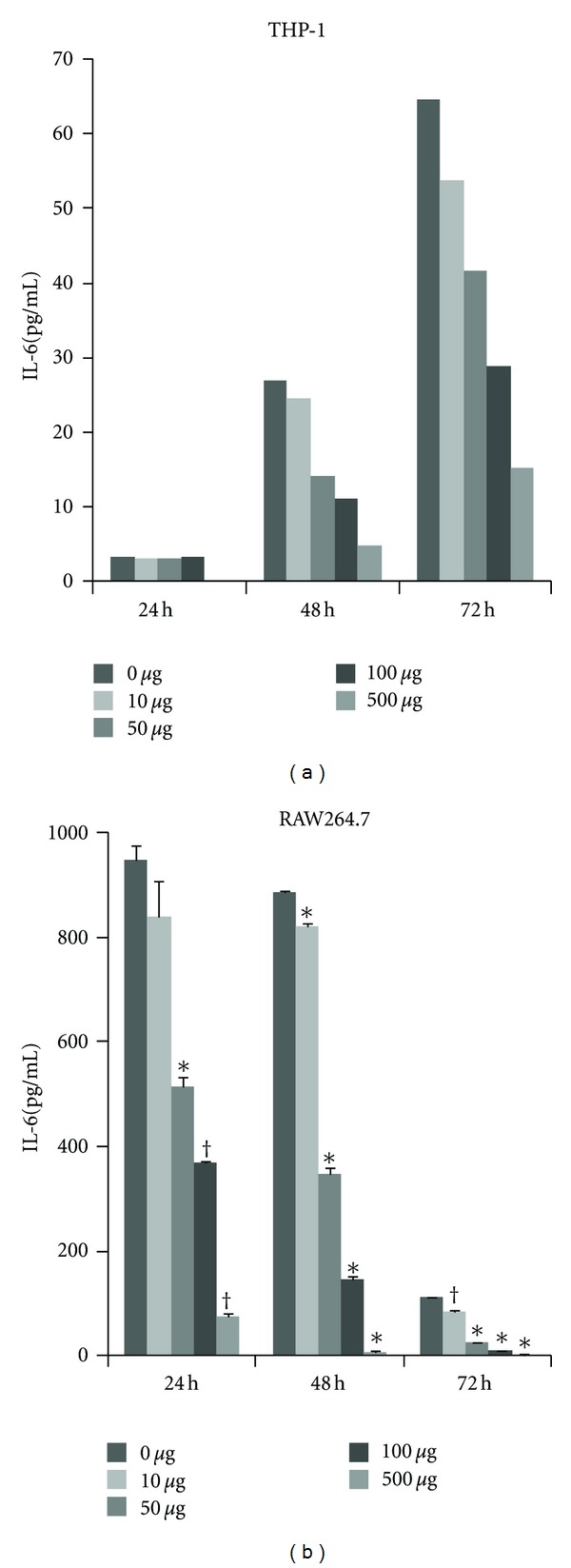
Effect of hochuekkito on IL-6 production from THP-1 or RAW264.7 macrophage cells in vitro. THP-1 (a) or LPS-stimulated RAW264.7 (b) cells were cultured in the absence of hochuekkito (0 *μ*g/mL) or presence of hochuekkito at different concentrations (10, 50, 100, and 500 *μ*g/mL). The culture supernatant was collected at 24, 48, and 72 hours, and the concentration of IL-6 secreted into the supernatant was quantitated by using multiplex suspension array for THP-1 (a) or ELISA for RAW264.7 (b), respectively. Multiplex suspension array was performed in single, and ELISA was performed in duplicate. Quantitative data are presented as means ± SD. Statistical significance was evaluated with unpaired Student's *t*-test. *Statistically significant difference compared with control (0 *μ*g/mL) at each time point; *P* < 0.01. ^†^Statistically significant difference compared with control (0 *μ*g/mL) at each time point; *P* < 0.05.

To examine the biological effects of hochuekkito on the secretion of proinflammatory cytokines by cancer cells or macrophages, we cultured C26 adenocarcinoma cells and 2 macrophage cell lines, THP-1 and RAW264.7, and treated them with hochuekkito. Treatment with hochuekkito at concentrations of 0, 10, 50, 100, and 500 *μ*g/mL did not affect the proliferation of C26 carcinoma cells, THP-1 cells, or RAW264.7 cells (data not shown). Concentration of the cytokines, including IL-6, TNF-*α*, IL-1, and IL-8, in the culture medium of THP-1 cells was analyzed using Multiplex Suspension Array. Among these cytokines, administration of hochuekkito suppressed the production of IL-6 by THP-1 cells in a dose-dependent manner at 48 and 72 h after administration (Figure 5(a)). On the basis of these findings, we also examined the inhibitory effect of hochuekkito on the production of IL-6 from RAW264.7 cells. Hochuekkito significantly suppressed the secretion of IL-6 from RAW264.7 cells (Figure 5(b)), but it did not inhibit the production of IL-6 from C26 adenocarcinoma cells (data not shown). These findings suggest that treatment with hochuekkito inhibited the production of IL-6 by macrophages without affecting cell proliferation.

## 4. Discussion

In our study, we used C26 tumor-bearing mice, a well-characterized animal model of cachexia and revealed that a Kampo formula, hochuekkito, ameliorated cachectic events such as reduction in carcass weight, food and water intake, gastrocnemius muscle weight, and fat tissue weight around the testes and decrease in serum triglyceride levels without any effect on tumor growth. Furthermore, treatment with hochuekkito reduced the serum IL-6 level and inhibited IL-6 production in macrophages in tissues surrounding tumors in mice with cachexia. Consistent with these findings, hochuekkito markedly inhibited the release of IL-6 from macrophage cells in vitro. These results strongly suggest that treatment with hochuekkito is effective and beneficial for the treatment of cancer cachexia by suppressing the production of proinflammatory cytokines, particularly IL-6 by macrophages. To the best of our knowledge, our study is the first report to reveal the anticachectic effect of hochuekkito in a well-established murine model of cachexia induced by C26 adenocarcinoma.

Recently, the biological effects of Kampo medicine have received much attention, and a number of studies support the efficacy and safety of several Kampo medicines in many fields [22]. For instance, hochuekkito has various biological effects such as increasing immunity [11, 12]. Interestingly, hochuekkito reduces the rhinovirus-induced secretion of IL-6, TNF-*α*, IL-1, and IL-8 from human tracheal epithelial cells in vitro [23]. In addition, treatment with hochuekkito decreases the serum levels of IL-6, TNF-*α*, and C-reactive protein and reduces body weight loss and improves the nutritional status of patients with COPD [13]. These basic and clinical indications strongly support our current findings that hochuekkito has the biological ability to inhibit the production of proinflammatory cytokines such as IL-6 by host cells and is effective and beneficial for the treatment of cancer cachexia. 

In the tumor microenvironment, host macrophages might be activated and produce proinflammatory cytokines [24]. The release of these cytokines by macrophages plays an important role in the progression of cancer cachexia [25]. Sophocarpine and matrine inhibit the production of IL-6 and TNF-*α* by the murine macrophage cell line, RAW264.7, and significantly attenuate the experimental cachexia induced by C26 adenocarcinoma in mice [17]. Thus, macrophage-derived cytokines, such as IL-6 and TNF-*α*, might be promising therapeutic targets for cancer cachexia. Although in our study, hochuekkito inhibited production of TNF-*α* by RAW264.7 cells (data not shown), level of serum TNF-*α* was below the lower limit of detection in our murine model. Thus, the implication of TNF-*α* in cancer cachexia in our murine model was not clear. In contrast, the serum IL-6 level was significantly upregulated in the cachexia murine model as compared to that in controls and was significantly decreased by the treatment with hochuekkito (Figure 3). Immunohistochemical analysis showed that IL-6 was strongly expressed in macrophages in tissues surrounding tumors in cachectic mice, and IL-6 production of macrophage was significantly inhibited by the treatment with hochuekkito, although level of IL-6 in cancer cells was not altered (Figure 4). These findings were consistent with our result that hochuekkito inhibited the release of IL-6 not from C26 carcinoma cells but from murine macrophage RAW264.7 cells in vitro. Therefore, future research should be directed towards understanding the mechanism by which hochuekkito suppresses the production of IL-6 by macrophages. 

Recently, much interest is focused on the role of lipase in cachexia [26, 27]. Activated lipase in adipose tissue breaks down the stored fat which consists predominantly of triglycerides, resulting in an increased serum triglyceride level and decreased fat deposition in the tissue in the initial step of cachexia. In our study, treatment with hochuekkito significantly decreased the serum IL-6 level and increased both the serum triglyceride level and weight of fat tissue in C26-bearing mice, although hochuekkito did not affect either the serum triglyceride level or weight of fat tissue in healthy controls (Table 1). No paper has been published about the effect of hochuekkito on lipase activity, and we did not examine the lipase activity in our cachectic murine model treated with hochuekkito. Therefore, the relationship between the increased triglyceride level and lipase activity in C26-bearing mice treated with hochuekkito was uncertain. However, our findings suggest that hochuekkito did not affect adipose triglyceride lipase activity because hochuekkito increased both the serum triglyceride level and fat deposition in cachectic mice but not in healthy controls. Several studies have reported that both the serum triglyceride level and weight of fat tissue were decreased in the terminal stage of cancer cachexia in mice [28, 29]. In this study, we evaluated the effect of hochuekkito on the cachectic state of tumor-bearing mice in the terminal stage, not in the initial stage. It has been also reported that administration of an NF-*κ*B inhibitor, dehydroxymethylepoxyquinomicin (DHMEQ), significantly decreased the serum IL-6 level in tumor-bearing cachectic mice, resulting in the improvement of both the epididymal fat weight and serum triglyceride level [30]. These previous findings are consistent with our result that treatment with hochuekkito significantly decreased the serum IL-6 level and increased both the serum triglyceride level and weight of fat tissue in C26-bearing mice in the terminal stage.

In conclusion, our study revealed that the Kampo formula, hochuekkito, ameliorated experimental cancer cachexia in mice and inhibited the production of macrophage-derived cytokines, especially IL-6. Our in vivo and in vitro studies provide valuable insights into the role of Kampo formula in the treatment for cancer cachexia and indicate that hochuekkito may be of great value in the future for patients with cancer cachexia, although further studies are necessary.

## Figures and Tables

**Table 1:** Changes in cachectic parameters in colon 26 adenocarcinoma-bearing mice.

Parameter	Tumor (−), Hochuekkito (−) (*n* = 5)	Tumor (−), Hochuekkito (+) (*n* = 5)	Tumor (+), Hochuekkito (−) (*n* = 4)	Tumor (+), Hochuekkito (+) (*n* = 4)
Carcass weight (g)	27.1 ± 0.7	26.2 ± 0.29	20.2 ± 2.3*	24.2 ± 2.9^††^
Gastrocnemius muscle (mg)	141.1 ± 10.3	128 ± 8.7	115.7 ± 6.9**	148.8 ± 8.9^†^
Fat tissue around testis (mg)	209.5 ± 14.4	226.5 ± 7.3	32.2 ± 55.7*	155.4 ± 77.9^††^
Hb (g/dL)	16.3 ± 1.2	15.4 ± 1	13.5 ± 0.9**	15.3 ± 1.6
Hct (%)	51.6 ± 3.2	48.2 ± 2.7	41.7 ± 2.1*	47.2 ± 5.1
Plt (×10^4^/*μ*L)	103.5 ± 9.7	115.6 ± 20.1	38.3 ± 8.7*	37.7 ± 13.6
Triglyceride (mg/dL)	117.2 ± 21.1	115 ± 14	16.8 ± 5.7*	63.8 ± 9.5^††^
Glucose (mg/dL)	176.9 ± 20.4	181.5 ± 22.3	61.7 ± 13.3*	126.6 ± 56.5

Colon 26 clone 20 adenocarcinoma cells were implanted subcutaneously to BALB/c mice, and hochuekkito was administered orally to mice for 15 consecutive days. Mice were sacrificed 15 days after the tumor inoculation, and blood samples were collected, and gastrocnemius muscle and fat tissue around testis were weighed at the time of killing. Hb: hemoglobin; Hct: hematocrit; Plt: platelets. Carcass weight was calculated as the difference between whole-body weight and tumor weight. Data represent mean ± S.D. Statistical significance was evaluated with Tukey's HSD after the one-way ANOVA.

*Significantly different from the group of tumor (−), hochuekkito (−); *P* < 0.01.

**Significantly different from the group of tumor (−), hochuekkito (−); *P* < 0.05.

^†^Significantly different from the group of tumor (+), hochuekkito (−); *P* < 0.01.

^††^Significantly different from the group of tumor (+), hochuekkito (−); *P* < 0.05.
